# Cell Type-Specific Regulation of Immunological Synapse Dynamics by B7 Ligand Recognition

**DOI:** 10.3389/fimmu.2016.00024

**Published:** 2016-02-04

**Authors:** Joanna Brzostek, Nicholas R. J. Gascoigne, Vasily Rybakin

**Affiliations:** ^1^Department of Microbiology and Immunology, Yong Loo Lin School of Medicine and Immunology Programme, National University of Singapore, Singapore, Singapore; ^2^Laboratory of Immunobiology, Rega Institute for Medical Research, KU Leuven, Leuven, Belgium

**Keywords:** Treg, PKC-theta, PKC-eta, CTLA-4, costimulation, immunological synapse, co-inhibition

## Abstract

B7 proteins CD80 (B7-1) and CD86 (B7-2) are expressed on most antigen-presenting cells and provide critical co-stimulatory or inhibitory input to T cells via their T-cell-expressed receptors: CD28 and CTLA-4. CD28 is expressed on effector T cells and regulatory T cells (Tregs), and CD28-dependent signals are required for optimum activation of effector T cell functions. CD28 ligation on effector T cells leads to formation of distinct molecular patterns and induction of cytoskeletal rearrangements at the immunological synapse (IS). CD28 plays a critical role in recruitment of protein kinase C (PKC)-θ to the effector T cell IS. CTLA-4 is constitutively expressed on the surface of Tregs, but it is expressed on effector T cells only after activation. As CTLA-4 binds to B7 proteins with significantly higher affinity than CD28, B7 ligand recognition by cells expressing both receptors leads to displacement of CD28 and PKC-θ from the IS. In Tregs, B7 ligand recognition leads to recruitment of CTLA-4 and PKC-η to the IS. CTLA-4 plays a role in regulation of T effector and Treg IS stability and cell motility. Due to their important roles in regulating T-cell-mediated responses, B7 receptors are emerging as important drug targets in oncology. In this review, we present an integrated summary of current knowledge about the role of B7 family receptor–ligand interactions in the regulation of spatial and temporal IS dynamics in effector and Tregs.

## Introduction

The adaptive immune system must distinguish between self and non-self in order to provide protection from pathogenic challenges while sparing the organism’s own tissues. Recognition of B7 ligands (CD80 and CD86, also known as B7-1 and B7-2, respectively) by co-stimulatory CD28 and co-inhibitory CTLA-4 (cytotoxic T-lymphocyte-associated protein 4, also known as CD152) receptors plays a critical role in regulation of effective self versus non-self discrimination. CD28 signaling is required for optimum proliferation and function of effector T cells, whereas CTLA-4 plays a critical role in negative regulation of immune responses, as it is required for turning off effector T cell signaling and regulatory T cell (Treg) development and suppressive functions. These opposing immunomodulatory roles of CTLA-4 and CD28 are of considerable clinical significance. CTLA-4 was the first immune checkpoint receptor targeted for cancer immunotherapy, and the anti-CTLA-4 antibody ipilimumab is used in the clinic for treatment of advanced melanoma (1). CD28 co-stimulatory function is also relevant for cancer immunotherapy, as chimeric antigen receptors (CARs) containing CD28 cytoplasmic regions have been shown to induce efficient T cell effector functions (2). However, targeting CD28 with the superagonistic monoclonal antibody TGN1412 was a tragic failure, when administration of the antibody during a phase I clinical trial induced severe systemic inflammatory responses in healthy volunteers (3). Therefore, a comprehensive understanding of expression patterns, signaling pathways, and functional roles of CD28 and CTLA-4 on effector and Treg subsets can have significant medical impact.

CD28 and CTLA-4 recognize their B7 ligands in the context of the cell-to-cell interface, termed the immunological synapse (IS), formed between a T cell and an antigen-presenting cell (APC). Receptor ligation at the IS leads to accumulation of interacting molecules at different regions of the synapse, forming distinct molecular patterns known as supramolecular activation clusters (SMAC) (4–6). The canonical mature T cell IS consists of a central SMAC (cSMAC) containing TCR (on the T cell) and pMHC (on the APC) molecules, surrounded by the peripheral SMAC (pSMAC) containing LFA-1 (on T cell) and ICAM-1 (on APC) adhesion molecules as well as F-actin. The outer ring of the IS, known as the distal SMAC (dSMAC) contains molecules with large ectodomains, such as CD45 and CD43. The SMAC regions contain smaller microdomains, known as microclusters (7). The IS is highly dynamic, with movement of TCR microclusters toward the center of the synapse, where they undergo endocytosis. Antigen recognition under physiological conditions does not always result in formation of this canonical IS structure; nevertheless this model provides a useful framework for understanding spatial dynamics of molecular interactions at the interface between T cell and APC membranes. The IS is the main site of immune receptor triggering and recruitment of signaling intermediates, leading to signal initiation and integration. B7 ligand recognition leads to distinct localization of CD28 and CTLA-4 receptors at the SMAC, modulation of cytoskeletal dynamics as well as recruitment of protein kinase C (PKC) isoforms to the IS. The effect of B7 ligand recognition on the IS dynamics is cell type specific, with effector T cells and Tregs displaying different CD28 and CTLA-4 localization, leading to differential recruitment of PKC-θ and PKC-η to the effector T cell and Treg synapses. This review presents a brief outline of the roles of CD28 and CTLA-4 in the immune system, followed by a more detailed discussion of CD28 and CTLA-4 localization patterns in the IS, and the consequences of B7 ligand recognition on IS structure and stability in T effector and Tregs.

## B7 Ligand Recognition: Structural Features and Expression Patterns

B7-1 and B7-2 (CD80 and CD86) molecules share a similar structure, consisting of one membrane-distal variable domain-like and one membrane-proximal constant domain-like immunoglobulin superfamily (IgSF) domain. Purified CD80 crystallizes in a dimeric form, and undergoes spontaneous homodimerization in solution (8), whereas CD86 crystalizes as a monomer (9). The two different oligomeric states of B7 were also observed using Forster resonance energy transfer (FRET) analysis on the surface of APCs, with CD80 present on the cell surface mainly in the form of dimers, and CD86 being monomeric (10, 11). CD80 and CD86 are expressed on dendritic cells (DCs), macrophages, and B cells, with CD86 displaying higher constitutive expression and more rapid upregulation after activation. B7 molecules are also expressed on activated mouse and human effector T cells (12–14). CD80 and CD86 bind to CTLA-4 with significantly higher affinity than to CD28. CD80 is a stronger ligand, with K_D_ 0.2 μM for CTLA-4 and 4 μM for CD28 interaction, whereas the K_D_ for CD86 binding to CTLA-4 is 2 and 20 μM for CD28 (15).

CD28 monomers consist of a V-like IgSF extracellular domain, transmembrane regions, and a short cytoplasmic tail with no enzymatic activity. CD28 is expressed on the cell surface as a glycosylated, disulfide-linked homodimer of 44 kDa chains. In adult humans, CD28 is constitutively expressed on approximately 80% of CD4+ and 50% of CD8+ T lymphocytes. Loss of CD28 expression, most marked in the CD8 compartment, has been observed in humans during aging and autoimmune diseases (16–18). CD28 is expressed on all mouse T cells, and it is not downmodulated during aging (19). Repeated *in vitro* antigenic stimulation (20, 21) and exposure to common-γ chain cytokines or type I interferons (22) leads to downregulation of CD28 expression on human T cells. However, *in vivo* antigenic stimulation has been reported to increase CD28 surface levels on mouse T cells (23).

**Figure 1:**
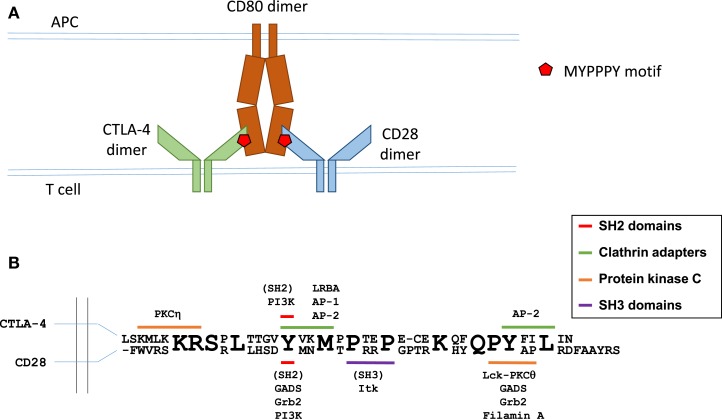
**Molecular interactions in B7 ligand recognition**. **(A)** Schematic representation of CD28 and CTLA-4 binding to the B7 ligands. **(B)** Schematic representation of the cytoplasmic regions of CTLA-4 (top sequence) and CD28 (bottom sequence). Known interaction partners of CTLA-4 are shown above and of CD28 below the alignment, and the motifs implicated in these interactions are color coded as indicated. Figure based on Hou et al. (40), Isakov and Altman (41, 42), Margulies (43), Schneider and Rudd (36), Sharpe and Freeman (44), and Stamper et al. (45).

CTLA-4 shares structural similarity with CD28, forming homodimers of V-like IgSF monomers. CTLA-4 contains a 36-amino-acid-long cytoplasmic tail with no enzymatic activity. CTLA-4 is not expressed on the surface of resting effector T cells (24, 25), but is expressed constitutively in Tregs (26) under control of Foxp3 and NFAT (27–29). In both conventional T cells and Tregs, surface CTLA-4 is continuously endocytosed via a clathrin- and dynamin-mediated pathway, and recycled to the plasma membrane (30–34). Activation of effector and Tregs leads to upregulated levels of CTLA-4 on the cell surface. CTLA-4 internalization is mediated by the heterotrimeric adapter protein AP-2 (30, 34, 35) [regulation of CTLA-4 trafficking is the subject of an excellent recent review in Ref. (36)], whereas CTLA-4 trafficking from the trans-Golgi network to the cell surface involves formation of a multimeric complex consisting of transmembrane adapters TRIM and LAX, as well as small GTPase Rab8 (37, 38). CTLA-4 present in recycling endosomes is protected from lysosomal targeting through interaction between LRBA protein (lipopolysaccharide-responsive and beige-like anchor protein) and CTLA-4’s tail region (39). Since its lysosomal degradation involves interaction with another clathrin adaptor complex AP-1 that binds to the same tyrosine-based motif (Y201) of CTLA-4 as LRBA (35) (the interaction motifs in CTLA-4 cytoplasmic region are summarized in Figure 1), it has been suggested that the binding of LRBA may prevent interaction with AP-1 and thereby protect the protein from degradation (39).

Both CTLA-4 and CD28 rely on the amino acid motif MYPPPY in the vicinity of Y139 in human CTLA-4 and Y123 in CD28 for binding to the B7 proteins (46–48). Importantly, despite the identical amino acid sequence of the interaction site, CTLA-4 and CD28 are capable of effectively discriminating between B7 proteins. A key study from the Allison lab (48) reported that the binding of a B7 ligand was critical for the concentration of CTLA-4 at the IS and contributed to the concentration of CD28, and that CD86 was a preferred ligand for CD28 and CD80 for CTLA-4. Antigen-pulsed B cells expressing CD80 effectively concentrated CTLA-4 at the synapse. Furthermore, in synapses formed by B cells expressing only CD80, there was evidence for competition between CTLA-4 and CD28 for ligand binding, as CD28 accumulation was reduced even further when CTLA-4 was present at the IS. Conversely, peptide-pulsed B cells expressing only CD86 strongly increased the accumulation of CD28 at the synapse, but failed to recruit CTLA-4 (48).

## CD28 in Regulation of the Immune Response

CD28 is the prototypic co-stimulatory molecule, and CD28 ligation leads to enhanced cytokine production, cell survival, and proliferation of effector T cells. The critical role of CD28-mediated signaling in optimum T cell responses is demonstrated by the T cell effector functions afforded to second-generation CARs containing cytoplasmic regions of CD28 and CD3ζ, but not by first-generation CARs lacking CD28 sequences (2). The cytoplasmic region of CD28 contains two main signaling motifs (summarized in Figure 1): a proximal YMNM motif and a distal proline-rich PYAP motif (49). The YMNM motif mediates phosphatidylinositol 3-kinase (PI3K) binding (50–52), leading to Akt activation; YMNM can also bind to GRB2/GADS adaptor proteins (51, 53) and the PYAP motif binds to Lck (54), filamin A, and GRB2/GADS (53, 55). The YMNM motif is followed immediately by another poly-proline motif PRRP, reported to bind the kinase Ikt (56). Analysis of knock-in mutant mice revealed that the PYAP motif is critical for IL-2 production and proliferation *in vitro*, as well as for *in vivo* antibody production and germinal center formation (57), whereas YMNM plays a role in augmenting T cell proliferation (58). Interestingly, knock-in T cells with both their YMNM and PYAP motifs mutated display less severe activation defects than CD28-deficient T cells, suggesting some functional role for the PRRP motif and/or yet unidentified cytoplasmic sequences.

CD28 is required for the thymic generation and peripheral maintenance of a functional Treg population. CD4+ Foxp3+ Tregs are key negative regulators of T cell-mediated immunity and are required for the control of spontaneous responses to self through several mechanisms (59, 60). Contact-mediated suppression relies on CTLA-4 interactions with its ligands and is discussed in detail below. Bystander suppression is mediated by suppressive cytokines, mainly IL-10 (61) and TGF-β (62) produced by activated Tregs, and by induction of cytokine starvation in target cells by IL-2 clearance (63). B7 ligand recognition plays an important role in Treg development and function, summarized in Table 1. In CD28-deficient NOD mice, the percentage of peripheral Tregs is strongly reduced (64). Similar reductions are observed in NOD mice lacking both CD80 and CD86, leading to the conclusion that the B7–CD28 interaction is required for the formation of the full Treg repertoire. The reduction in the percentage of Tregs in NOD mice treated with B7-blocking CTLA-4-Ig correlates with a higher incidence of spontaneous autoimmune diabetes (64). Subsequent analysis revealed that Treg deficiency in CD28^−/−^ mice can be traced back to thymic development. The percentage of Treg precursors among thymic CD4 single-positive cells is significantly reduced in CD28^−/−^ mice as well as in NOD mice injected with anti-CD80 and CD86 antibodies (65), and in B7 double knockout mice (66). Peripheral homeostatic expansion of Tregs – but not effector T cells – in normal syngeneic hosts is also strongly suppressed by anti-CD80 and CD86 antibodies (66). A mechanistic explanation for the thymic requirement for CD28 was proposed by Tai et al. (67) who examined the consequences of CD28 deletion in a TCR-transgenic model. Mice expressing the AND TCR and its agonist ligand, a pigeon cytochrome *c* peptide, were found to effectively induce thymic Tregs only in the presence of CD28. This means that while a strong selection signal through TCR is indeed required (68), it is not sufficient for the full initiation of the agonist selection program leading to the generation of Tregs, and that a co-stimulatory signal from mTEC-expressed B7 molecules through CD28 is also required. It is noteworthy that a small proportion of regulatory phenotype T cells were still generated in the absence of CD28 but these cells lacked suppressive capacity (67). Earlier data from the same group indicated that CD28 is also required for the deletion of thymocytes by negative selection (69, 70).

**Table 1:** **B7 ligand recognition in Treg synapse formation and suppressive functions**.

Surface interactions	Biological significance for Tregs	Reference
CD28–B7	Motility Tonic signals necessary for survival	Lu et al. (160), Thauland et al. (163) Zhang et al. (71)
CD28–B7	Antigen recognition Motility stop signal	Apostolou et al. (176), Jordan et al. (68), Knoechel et al. (177), Walker et al. (178)
		Lu et al. (160), Thauland et al. (163)
TCR–pMHC	Synapse formation and stabilization	Onishi et al. (161)
	Activation	Schmidt et al. (179), Zhang et al. (71)
LFA-1–ICAM-1	Proliferation	Walker et al. (178), Zheng et al. (155)
	Surface accumulation of CTLA-4	Catalfamo et al. (170)
CTLA-4–B7	Synapse stabilization	Onishi et al. (161), Zanin-Zhorov et al. (151)
TCR–pMHC		
LFA-1–ICAM-1	Contact suppression	Kong et al. (152), Qureshi et al. (146)

A study using mice in which CD28 was selectively deleted in cells expressing Foxp3 (Cd28-ΔTreg), reported only a minor decrease in the percentage of thymic Treg precursors (71). This is in line with previous observations that CD28 is involved in the generation of early, Foxp3-negative Treg precursors (72). However, in stark contrast to earlier studies, homeostatic expansion of Tregs in the periphery was reported to occur independently of CD28 (73). Tregs from CD28-ΔTreg mice displayed reduced suppressive capacity, and consequently CD28-ΔTreg animals developed spontaneous autoimmunity (71). Costimulation through CD28 is required for *in vivo* expansion of Tregs in the presence of TCR stimulation and IL-2 (74). CD28 stimulation is also required for the conversion of naïve CD4 T cells into Tregs *in vivo* (75, 76) and *in vitro* (77).

## CTLA-4 in Regulation of the Immune Response

CTLA-4 is a critical negative regulator of the immune response. Germline CTLA-4 knockout in mice results in massive lymphoproliferation (78), and is lethal at 3–4 weeks of age (78, 79). The peripheral T cell profile in these animals is strongly skewed toward CD4 cells that rapidly proliferate in a CTLA-4-Ig-sensitive manner – indicating the dependence on B7–CD28 interaction – and infiltrate non-lymphoid tissues (78, 80). Introduction of CTLA-4-sufficient Tregs reverts the lymphoproliferative disorder and prevents early lethality in CTLA-4 knockout mice (81), whereas blocking of CTLA-4 on Tregs completely abrogates their suppressive function (62, 66, 82). CTLA-4-deficient Tregs are unable to control lymphopenia-driven homeostatic expansion of conventional CD4 cells (83). Importantly, interaction between CTLA-4 and B7 expressed on effector T cells was found to be dispensable for the control of the latter in mixed bone marrow chimera experiments as both B7^−/−^CTLA-4^−/−^ and B7^+/+^CTLA-4^−/−^ effector T cells were efficiently suppressed by CTLA-4-sufficient Tregs (66). B7 expression is also not required on Tregs themselves (66). These data indicate that B7 expressed on a cell subset distinct from effector and Tregs mediates interactions with Treg-expressed CTLA-4 and immune suppression. CTLA-4-deficient Tregs are characterized by similar expression of CD25, PD1, GITR, and of suppressive cytokines IL-10 and IL-35 (83). Foxp3 promoter-controlled deletion of CTLA-4 in Tregs resulted in lymphoproliferative disease and tissue infiltration, and was lethal at ~7–8 weeks of age [i.e., somewhat delayed compared to germline knockout Ref. (84)]. Similarly to Foxp3-driven CD28 deficiency, thymic development of Tregs was normal, as was their survival in the periphery. However, cells lacking CTLA-4 were unable to control proliferation of target cells stimulated by anti-CD3 antibody and DC, and to induce tumor rejection (84).

Unlike CD28, CTLA-4 is not required for Treg development in the thymus. CTLA-4 is expressed by a subset of thymocytes predominantly residing at the corticomedullary junction (85) and is strongly upregulated upon induction of negative selection (86). There is no requirement for CTLA-4 expression to initiate central Treg development and peripheral expansion, as CTLA-4 knockout mice exhibit elevated percentage of Tregs and increased Ki67 expression, indicative of their active proliferation (87). Moreover, deletion of CTLA-4 in TCR-transgenic mice increases the frequency of Foxp3-positive Treg precursors in the thymus and leads to the formation of a specific population of Foxp3-positive DP thymocyte subsets in the thymic cortex (85). However, CTLA-4 can play a role in formation of the induced Treg population, as CTLA-4 has been shown to induce expression of Foxp3 and Treg conversion in the intestine (88).

## CD28 and Immunological Synapse Architecture in Effector T Cells

CD28 shows a unique cSMAC localization pattern that is important for its efficient co-stimulatory functions. CD28 co-localizes with TCR microclusters at the earliest observable time-point after agonist pMHC recognition (89, 90), and the early accumulation of CD28 at the IS shows similar kinetics and localization as the TCR complex. In a mature IS, CD28 is present at the cSMAC, but segregates away from TCR (90, 91). This segregation of CD28 from TCR at the IS is required for optimum T cell activation, as shown in a study comparing different anti-CD3 and CD28 micropatterns on planar stimulatory surfaces (92). The spatial separation of TCR and CD28 at the mature IS is regulated by localization of CD28 ligands, as full length CD80 separates from TCR at the IS, but CD80 with deleted cytoplasmic region localizes with TCR (93). Moreover, the tailless CD80 molecule does not provide an optimum co-stimulatory signal and does not show efficient accumulation at APC: T cell contact interface (94, 95). This suggests a role for B7 interactions with cytoskeleton and/or other cytoplasmic components in regulation of IS architecture. CD28 recruitment and maintenance at the synapse requires both CD28 and TCR ligand binding (90, 96). CD28 accumulation at the synapse has been shown to be independent of antigenic pMHC affinity to TCR, with weak and strong agonist pMHC complexes inducing similar levels of CD28 recruitment (97). The role of CD28-mediated signaling and interactions in regulation of CD28 localization at the synapse is somewhat controversial, with a report indicating unperturbed IS localization of CD28 with mutated or deleted cytoplasmic region (90), whereas another study observed impaired IS localization of CD28 with deleted cytoplasmic domain or with a mutation at Y188 within the CD28 PYAP motif (96).

CD28 ligation has been shown to induce rapid internalization of the receptor, with half of the endocytosed fraction degraded in lysosomes and half recycled back to the cell surface (98). CD28 downregulation depends on PI3K (73), with preferential endocytosis of CD28 molecules associated with PI3K (98). CD28 is endocytosed via clathrin-coated pits, and this process requires coupling of WASP to PI3K and CD28 via sorting nexin 9 (73). CD28 downregulation from the synapse can also be influenced by stoichiometry of its B7 ligands (11). FRET analysis of B7 fluorescent protein fusions demonstrated that CD80 is present at the cell surface as a mixed population of dimers and monomers, with CD86 predominantly present in monomeric form (10). Experimental increase in CD80 dimerization resulted in enhanced T cell: APC conjugate formation and more sustained accumulation of Lck and PKC-θ at the IS (11).

Co-stimulatory signals play a critical role in regulation of cytoskeleton dynamics at the IS during T cell interaction with APC (summarized in Table 2). CD28 ligation induces movement of actin cytoskeleton toward the IS (99), and CD28 engagement is required for sustained actin accumulation at the IS (100). CD28 stimulation alone leads to actin polymerization and recruitment of actin at the IS (101). CD28 signaling is important in multiple pathways involved in actin filament nucleation, elongation, and depolymerization. The guanine nucleotide exchange factor Vav1 controls the activity of small Rho GTPases Cdc42 and Rac1 that regulate actin polymerization activity of WASP and WAVE2, respectively. WASP and WAVE2 are actin nucleation-promoting factors that, together with the Arp2/3 complex, facilitate formation of new actin filaments.

**Table 2:** **CD28 in regulation of cytoskeleton dynamics at the immunological synapse**.

Cytoskeletal regulator	CD28-induced modification	Effect on cytoskeleton	Reference
Vav1	Phosphorylation, leading to activation	Vav1 controls activity of small Rho GTPases Cdc42 and Rac1 that regulate actin polymerization activity of WASP and WAVE2, respectively	Nunes et al. (102), Raab et al. (104), Salazar-Fontana et al. (103), Schneider and Rudd (106)
Filamin A	Direct interaction with CD28, phosphorylation	Filamin A has a role in actin crosslinking	Muscolini et al. (111), Tavano et al. (108)
Cofilin	Dephosphorylation, leading to activation	Actin severing protein. Blocking cofilin–actin interaction reduces T cell:APC conjugation	Lee et al. (119), Wabnitz et al. (120)
Rltpr	Unknown	Actin-uncapping protein. Wild-type Rltpr is required for CD28-dependent costimulation, but this seems to be independent of its actin-uncapping function	Liang et al. (113)
CapZIP	Phosphorylation	Actin-uncapping protein. CapZIP is required for CD28-dependent costimulation, but its effect on T cell cytoskeleton are unknown	Tian et al. (110)

CD28 ligation induces tyrosine phosphorylation of Vav1 (102), and CD28-dependent actin remodeling requires Cdc42 (103) and Rac1 (104). The molecular interactions linking CD28 to Vav1 phosphorylation are not yet fully elucidated. CD28-dependent Vav1 phosphorylation has been shown to require binding of the adaptor protein GRB2 to CD28 (105, 106), but a recent report provided evidence for GRB2-independent Vav1 binding to CD28 and a role of PIP5K1A (phosphatidylinositol 4-Phosphate 5-Kinase α) and Vav1 cooperation in regulation of actin, downstream of CD28 (107). Jurkat cells expressing CD28 with mutated C-terminal PYAP motif, important for GRB2 binding, failed to recruit Vav1 to the IS or to rearrange actin after CD28 ligation (107); however, Vav1 phosphorylation in response to CD28 ligation was not assessed in this study, and in another report the PYAP motif was shown to be dispensable for CD28-dependent Vav1 phosphorylation (108). The Arp2/3 actin nucleation complex cooperates with filamins, large multidomain proteins with a role in actin crosslinking (109), to establish actin structure. Filamin A is phosphorylated (110) and recruited to the IS (108) after CD28 ligation in T cells, with the PYAP motif on the cytoplasmic region of CD28 mediating the interaction with filamin A (108, 111). Knockdown of filamin A expression did not affect CD28-mediated Vav1 phosphorylation, but reduced Cdc42 activity and impaired CD28-mediated costimulation (108). However, changes in actin structure or dynamics at the IS of filamin A knockdown cells have not been reported. Moreover, knocking down filamin A did not impair ezrin accumulation at the IS (108).

Actin filaments contain a fast growing barbed end, which can be bound to actin capping protein. Capping protein binding to the barbed end prevents addition of new actin subunits, limiting the filament elongation. Actin capping and subsequent actin polymerization can be regulated by actin-uncapping proteins (112). An actin-uncapping protein Rltpr is required for CD28-dependent costimulation (113), and Rltpr colocalizes with CD28 in CD80-dependent signaling microclusters (113), suggesting a role of Rltpr in CD28-mediated actin rearrangement at the synapse. However, a direct role of Rltpr for CD28-dependent actin modification is unclear. Rltpr does not immunoprecipitate with CD28 (113) and is not phosphorylated after CD28 ligation (110). Moreover, an Rltpr mutation that abolishes CD28-mediated costimulation does not impair Rltpr’s actin-uncapping ability or CD28-dependent actin rearrangements at the synapse (113). The Rltpr mutation that reduced CD28-dependent costimulation abrogates CD28-dependent recruitment of PKC-θ and Carma1 to the IS (113) through a yet unidentified molecular mechanism, suggesting that Rltpr acts as an adaptor at the IS independently of its actin-uncapping functions. A recent phosphoproteomic screen identified actin-uncapping CapZIP as part CD28 signaling network (110). Importantly, CapZIP is required for CD28-dependent costimulation of cytokine production (110). However, it has not yet been reported if CapZIP can directly interact with CD28 and if it is required for CD28-dependent changes in actin dynamics. In summary, the current evidence suggests that CD28-dependent signaling may regulate actin capping through actin-uncapping proteins CapZIP and potentially Rltpr.

CD28 signaling regulates actin dynamics through control of activity of the actin-severing protein cofilin. Cofilin is a ubiquitously expressed 19 kDa protein that cleaves actin filaments, thus, promoting actin depolymerization, but also creating new barbed ends for filament elongation (114). Cofilin’s actin binding capacity is negatively regulated by its phosphorylation at serine 3 (115, 116), and binding to phospholipids (117). Blocking cofilin interaction with actin reduces T cell proliferation and cytokine production, as well as conjugation with APCs (118). In resting human T cells, cofilin is present mainly in the inactive phosphorylated form, and CD28 or CD2 signal together with TCR, but not TCR signal alone, induces cofilin dephosphorylation and actin binding (119, 120). The precise sequence of signaling events linking CD28 ligation to cofilin activation is unknown. Cofilin is dephosphorylated by serine phosphatases PP1 and PP2A (121), and CD3/CD28-induced cofilin dephosphorylation requires Ras (120). Additionally, CD28 may regulate cofilin activity through control of levels of membrane phospholipids (114).

There is strong evidence that CD28-dependent regulation of actin dynamics is important for the effector T cell functions. CD28 enhances T cell:APC conjugate formation *in vitro* (122, 123). Knock-in mice with mutated PYAP motif show reduced IL-2 production and proliferation *in vitro*, and impaired *in vivo* antibody production and germinal center formation (57). This could be a result of impaired cytoskeletal rearrangement, as the PYAP motif is implicated in Vav1 and filamin A recruitment. However, the effects of PYAP mutations on cytoskeletal dynamics and synapse stability have not yet been reported for primary T cells, and this motif is also important for binding to Lck (54), GRB2/GADS (53, 55), and PKC-θ (124), as discussed below. Analysis of a mouse mutant with inducible inhibition of Csk, a negative regulator of Src family kinases, strongly suggested that CD28-dependent actin remodeling is critical for initiation of full TCR signal in thymocytes (125). However, thymocytes from PYAP mutant knock-in mice do not show obvious phenotypic defects (57), suggesting that CD28-independent pathways can regulate actin cytoskeleton dynamics during thymocyte development.

## CD28 and Regulation of PKC-θ Localization at the Effector T Cell IS

CD28 plays a critical role in regulation of the IS localization of the novel protein kinase C (nPKC) isoform PKC-θ (summarized in Table 3). The PKC family consists of 10 serine/threonine kinase isoforms, with important roles in regulation of multiple cellular processes in different cell types. All nPKC isoforms (PKC-θ, PKC-δ, PKC-ϵ, and PKC-η) require diacylglycerol (DAG), but not Ca^2+^, for activation, and are expressed in T cells and play multiple roles in regulation of T cell signaling and effector functions (126). Central localization of PKC-θ is one of the hallmarks of the mature effector T cell IS. A seminal study by Monks at al. identified PKC-θ as the only PKC isoform recruited to effector T cell IS (127). However, more recent studies show that PKC-η and PKC-ϵ are also recruited (128–130), with some evidence that their recruitment precedes that of PKC-θ (129). PKC-ϵ and PKC-η display homogeneous distribution over the entire synapse, whereas PKC-θ displays discrete cSMAC localization contained within the peripheral actin ring (128–132).

**Table 3:** **Molecular determinants of PKC-θ localization at the immunological synapse**.

Interaction/activity	Molecular determinants	Effect on immunological synapse	Reference
PKC-θ–CD28	Polyproline motif within the PKC-θ V3 hinge region and PYAP motif in CD28; Lck-mediated interaction	PKC-θ V3 hinge and CD28 PYAP motif are required for CD28 cSMAC localization	Kong et al. (124)
PKC-θ–CD28	Sumoylation of PKC-θ at lysines 325 and 506	Abrogated PKC-θ sumoylation reduces PKC-θ localization at the IS and its colocalization with CD28, induces colocalization of PKC-θ and filamin A at periphery of the IS	Wang et al. (139)
PKC-θ–DAG	C1 domains of PKC-θ	C1 domains mediate initial PKC-θ recruitment to the synaptic membrane, but they do not support PKC-θ central accumulation at the synapse	Basu et al. (134), Carrasco and Merida (136), Quann et al. (135)
PKC-θ kinase activity	Unknown, possibly through autophosphorylation at threonine 219 between the tandem C1 domains	PKC-θ kinase activity is required for its recruitment to the IS	Cartwright et al. (138), Thuille et al. (137)
Rltpr	Unknown, no interaction between Rltpr and PKC-θ has been detected	Wild-type Rltpr is required for PKC-θ and CARMA1 recruitment to cSMAC	Liang et al. (113)

An important study using lipid bilayers presenting antigen and co-stimulatory signal, and TIRF microscopy to examine PKC-θ localization at the effector T cell IS, revealed initial colocalization of PKC-θ with TCR/CD28 microclusters (90). This was followed by PKC-θ recruitment to the cSMAC, where it segregated, together with CD28, to TCR^low^ regions in the periphery of cSMAC (90). The initial stages of PKC-θ recruitment to the effector T cell IS do not depend on CD28 ligand binding, but CD28 ligation is required for sustained PKC-θ localization at the synapse and colocalization of PKC-θ with CD28 (90, 133). PKC-θ interacts with CD28 after PMA treatment (90) (which induces PKC activation) and TCR/CD28 stimulation (124).

The molecular determinants of PKC-θ synapse localization have been mapped to the V3 hinge region and C1 domains (132). nPKCs share a conserved structure, with an amino-terminal C2 domain, tandem C1 domains, and V3 hinge linked to a carboxyl-terminal kinase domain. PKC-θ interaction with CD28 and cSMAC localization requires a polyproline motif within the V3 hinge region (124), and V3 hinge regions from PKC-ϵ and PKC-η mediate their diffuse accumulation at the synapse (134). A carboxyl-terminal poly-proline motif (PYAP) in the CD28 cytoplasmic tail is required for its interaction with PKC-θ, with strong evidence suggesting that this is an indirect interaction mediated through Lck, with the Lck SH3 domain binding to the polyproline motif in PKC-θ V3 and the Lck SH2 domain binding a phosphorylated tyrosine within the CD28 PYAP motif (124). Tyrosine 188 within the PYAP motif was also identified as critical for CD28 and PKC-θ central synapse localization in an earlier study (96).

Additionally, C1 domains of PKC-θ also play a role in its synapse localization, through interaction with DAG at the synapse membrane (134, 135). C1 domains can mediate initial PKC-θ recruitment to the synaptic membrane (135), but they do not support PKC-θ central accumulation and retention and the membrane (136), and nPKC C1 domains are not sufficient to determine the respective synapse localizations of PKC-θ versus PKC-ϵ and PKC-η (134). Phosphorylation of PKC-θ threonine 219 (T219), in a hinge region between the tandem C1 domains, is required for PKC-θ localization at the IS (137). Moreover, sustained synapse localization is dependent on PKC-θ kinase activity (137, 138), most likely through a requirement for PKC-θ autophosphorylation at T219 (132, 137). PKC-θ recruitment to the IS also requires expression of wild-type Rltpr actin-uncapping protein (113). T cells from mice expressing an Rltpr mutant could not recruit PKC-θ to the IS (113). The precise role of Rltpr in the regulation of PKC-θ synapse localization is unknown but seems to be independent of Rltpr actin-uncapping function, and no direct interactions between Rltpr and PKC-θ have been observed (113).

A recent report identified a novel activation-dependent post-translational modification of PKC-θ that modulates CD28–PKC-θ interactions and IS architecture (139). TCR stimulation of resting murine and human T cells leads to conjugation of SUMO1 (small ubiquitin-like modifier) to PKC-θ lysine (K) 325 and K506 by SUMO E3 ligase PIASxβ (139). Importantly, TCR and CD28 costimulation resulted in stronger PKC-θ sumoylation than TCR stimulation alone. Sumoylation-resistant PKC-θ with mutated K325 and K506 residues showed reduced interaction with CD28 and filamin A, and diffuse localization at the membrane in the IS (139). Inhibiting PKC-θ sumoylation through PIASxβ knockdown or overexpression of a desumoylating enzyme also abrogated PKC-θ localization at the IS, and reduced its colocalization with CD28 (139). Wild-type PKC-θ segregated from filamin A at the IS, with mainly pSMAC localization of filamin A. Inhibition of PKC-θ sumoylation altered the IS architecture, inducing colocalization of PKC-θ and filamin A at the periphery of the synapse (139).

The localization of PKC-θ to the center of the IS is critical for its functions in effector T cells. Mutations of the poly-proline motif in the V3 region of PKC-θ reduced activation of primary effector CD4+ T cells (124). Critically, overexpression of murine V3 domain sequesters PKC-θ from CD28 and cSMAC in mouse CD4+ T cells, and reduces PKC-θ-dependent gene expression *in vitro*, as well as CD4+ Th2 and Th17 immune responses *in vivo* (124). Similarly, expression of sumoylation-resistant PKC-θ mutants, with impaired synapse localization, does not rescue defects in cytokine production, activation of PKC-θ dependent transcription factors, and Th2 differentiation of human T cells with downregulated expression levels of endogenous PKC-θ (139). Additionally, mutations in the CD28 PYAP motif, required for PKC-θ interaction with CD28 and for IS localization, severely impaired effector T cell functions *in vivo* (57). However, it must be noted that PKC-θ synapse localization seems to be inseparable from its interaction with CD28, and the observed functional effects of impaired PKC-θ synapse recruitment could also be caused by reduced CD28 interactions with PKC-θ, Lck, and/or filamin A.

## CTLA-4 Dynamics at the Effector and Treg IS

Recognition of B7 ligands by CD28 and CTLA-4 at the effector T cell IS leads to competitive displacement of CD28 and PKC-θ from its central region. In the absence of stimulation, regulatory and conventional T cells express similar levels of CD28, but CTLA-4 expression is significantly higher in unstimulated Tregs (71, 140, 141). TCR signaling induces polarization of both intracellular (142) and membrane pools of CTLA-4 toward the IS of effector T cells, and TCR signal strength determines CTLA-4 localization at the IS (97). CTLA-4 is recruited to the effector T cell cSMAC with delayed kinetics relative to that of TCR and CD28, segregates away from CD3^high^ regions and forms a ring-like structure (141). CTLA-4 recruitment to and stabilization at the IS depends on its ligand binding, but occurs at both high and low B7 ligand densities (141). Critically, recruitment of CTLA-4 to the IS influences CD28 localization, due to competition for ligand binding. At high ligand densities, CTLA-4 recruitment leads to exclusion of CD28 from the cSMAC and its accumulation outside the pSMAC (141). At low ligand densities, CTLA-4 prevents formation of CD28 clusters at the T effector IS (141). Importantly, CTLA-4-mediated displacement of CD28 from the cSMAC leads to impaired synaptic localization of PKC-θ (141). CTLA-4 ligation has also been reported to reduce the size of T cell: APC contact interface and to reduce ZAP70 microcluster formation (143).

The localization of CTLA-4 in the T effector synapse depends on the molecular dimensions of the extracellular region of the protein, as CTLA-4 molecules with elongated ectodomains failed to accumulate at cSMAC despite unimpaired ligand binding (141). However, it has not been reported if CTLA-4 with elongated ectodomains affected CD28 clustering at the synapse, and it is not known whether similar dimensions of CD28 and CTLA-4 receptor–ligand complexes are important for efficient regulation of co-stimulatory signal and/or competition for ligand binding at the synapse. The matching sizes of activating and inhibitory receptor–ligand complexes are critical for signal integration and regulation of NK-cell functions (144, 145), and it is plausible that a similar requirement exists for co-stimulatory and co-inhibitory signaling in effector T cells.

A molecular mechanism for CTLA-4 involvement in the downregulation of CD80/CD86 has been established in the seminal work by Qureshi et al. (146). Using co-cultured CHO cells expressing either human CTLA-4 or GFP-tagged human CD86, they observed transfer of GFP signal into CTLA-4 expressing cells, and its accumulation in the endolysosomal system, indicative of CD86 trans-endocytosis. Endocytosis-deficient CTLA-4 failed to induce trans-endocytosis of CD86-GFP and resulted in the accumulation of CD86 at cell contacts. These findings were confirmed using purified human Tregs incubated with DC where CD86 expression on the surface of the DC was reduced in the presence of Tregs but not effector T cells, and TCR stimulation increased the rate of trans-endocytosis (146). The most direct consequence of reduction of B7 proteins on the surface of APC is manifested in fewer and less prolonged interactions between APC and effector T cells (147, 148) reduced PKC-θ recruitment and activation in these cells (149) and, consequently, repression of IL-2 production by effector T cells (150). Recently, it has been shown that surface expression of CTLA-4 on effector T cells is sufficient for downregulation of CD86 expression from APCs (40).

**Figure 2:**
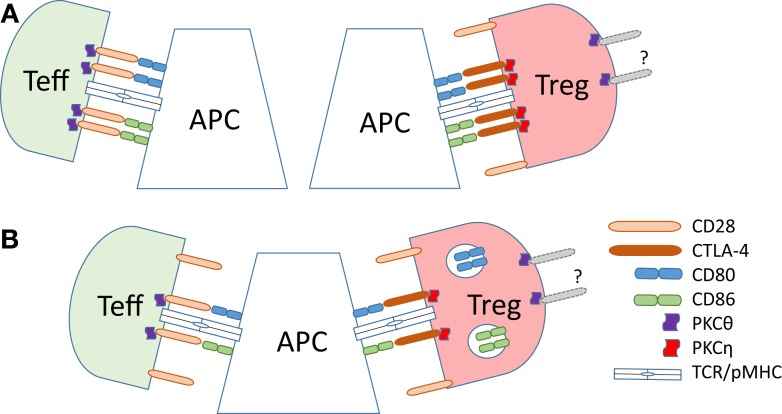
**Dynamics of B7 ligand recognition at effector and Treg IS**. **(A)** B7 (CD80 or CD86) ligation leads to accumulation of CD28 and associated PKC-θ at the T effector cell IS. High-affinity B7 binding by CTLA-4 on Tregs leads to accumulation of CTLA-4 and the associated PKC-η at the Treg IS, and exclusion of CD28 and PKC-θ from the IS. **(B)** CTLA-4 ligand binding in Tregs results in the trans-endocytosis of the B7 ligands. This reduces the amount of the B7 ligands on the surface of the APC, leading to reduced co-stimulatory signals delivered to effector T cells.

Tregs display radically different synapse localization of CD28 and PKC-θ than effector T cells (summarized in Figure 2). In a stimulating planar lipid bilayer system, the recruitment of CD28 to the Treg IS is barely detectable, whereas CTLA-4 is recruited robustly, in stark contrast to conventional CD4 T cells (141). Displacement of CD28 from Treg synapses by CTLA-4 coincides with the absence of PKC-θ clusters in the cSMAC zone of Treg synapses. Similarly, the switch of developmental program during the *in vitro* conversion of naïve CD4 T cells into Tregs results in a loss of PKC-θ signal at the synapse. Correct localization of CTLA-4 to the IS is functionally important, as elongation of the extracellular domain of CTLA-4 resulted in a loss of its concentration in the synapse and reduction of suppressive activity of Tregs (141). In a lipid bilayer system, addition of CD80 or ICAM-1 to the bilayer increases the recruitment of PKC-θ to the synapse in both effector and Tregs, but stimulation through TCR strongly decreases the recruitment in Tregs (151). Reduction of PKC-θ activity results in increased Treg proliferation and elevated suppressive capacity (151).

An important insight into the signaling mechanism downstream of CTLA-4 recruitment to the Treg synapse was provided in a recent study by Kong et al. (152) which identified PKC-η as the only PKC isoform physically interacting with CTLA-4. In Tregs, a phosphorylated form of PKC-η binds constitutively to CTLA-4. PKC-η localizes to the IS in close proximity to the TCR (152). Interaction between PKC-η and CTLA-4 was found to be critical for Treg function. Phosphorylated serine residues S28, S32, and S317 of PKC-η are responsible for the interaction with CTLA-4, and loss of S28 or S32 results in a strong inhibition of some Treg suppressive functions. The importance of PKC-η was further emphasized by the finding that, although PKC-η-deficient Tregs expressed normal levels of functional LFA-1 required for the stabilization of contacts with APC, they showed a marked decrease in their ability to continuously clear CD86 from the APC surface. While CD86 clearance on first contact with APC was unaffected by the loss of PKC-η, the reduction of CD86 on reintroduced APC was substantially delayed (152). These findings suggest that PKC-η is not directly involved in CTLA-4-induced trans-endocytosis, and that a feedback signaling mechanism from PKC-η may be required for the recycling of CTLA-4 from the endolysosomal system. It remains to be elucidated whether or not the amount of surface-expressed CTLA-4 is reduced and its intracellular retention or lysosomal degradation is accelerated in the absence of PKC-η. In an earlier study, a deletion of amino acids 191–223 of the intracellular domain of CTLA-4 did not substantially affect the *in vitro* suppression of target T cell proliferation in the presence of CD3 crosslinking antibody and APC, or *in vivo* suppression in a colitis model (26). Kong et al. have shown that this deletion-mutant of CTLA-4 retains its association with PKC-η, suggesting that the remaining cytoplasmic portion (amino acids 182–191) of CTLA-4 is sufficient for suppressive signals using PKC-η.

Responses to CD80 and CD86 signals in effector T cells are largely similar (153, 154). By contrast, addition of anti-CD80 antibody or CTLA-4 Fab fragments to the co-culture of target T cells, DC, and prestimulated Tregs ablated the suppressive function of the latter, whereas addition of anti-CD86 or anti-CD28 antibodies increased suppression to the same degree (155). Furthermore, blocking CD86 inhibited DC-induced division of Tregs, whereas blocking CD80 enhanced division (155). These data suggest that, in contrast to effector T cells, Tregs can effectively discriminate between CD80–CTLA-4 and CD86–CD28 signals.

## CTLA-4 in Regulation of Regulatory and Effector T Cell Synapse Stability and Cell Motility

A growing body of evidence clearly suggests a role for CTLA-4 in regulation of synapse stability, duration of conjugation with APC and overall motility in Treg and Teff cells. Anti-CTLA-4 blocking antibody treatment has been shown to increase effector T cell motility *in vitro* (122, 156) and *in vivo* (157–159). Importantly, it has been reported that CTLA-4 ligation has different outcomes for synapse stability and motility of regulatory versus effector T cells (122, 160).

Regulatory T cells form a more stable IS than effector T cells, and this enhanced synapse stability has been implicated in CTLA-4-dependent downregulation of B7 cell surface ­expression by Tregs. In mixtures with conventional CD4 T cells of same specificity, TCR-transgenic Tregs preferentially bind to DCs and exclude conventional cells (161). Similarly, in a planar lipid bilayer system, Tregs form a more long-lived IS than do effector T cells of the same specificity (151). Addition of blocking CTLA-4 antibody does not overrule the competitive advantage of Tregs, but loss of LFA-1 results in its reversal, indicating that LFA-1 is at least partially responsible for the preferential binding of Tregs (161). Stimulated TCR-transgenic Tregs specifically reduce expression of both CD80 and CD86 on DC and to lesser degree B cells (84, 87, 161, 162). In the absence of stimulating peptide, the B7 molecules are not downregulated. CTLA-4-deficient Tregs as well as wild-type cells in the presence of CTLA-4 blocking antibody do not reduce B7 expression, and neither do Tregs from LFA-1^−/−^ mice (84, 161), indicating that the formation of a stabilized, LFA-1 dependent, Treg-APC IS is important for the B7 downregulation.

B7 ligand recognition can modulate Treg motility. Tregs migrate rapidly on non-stimulating bilayers but slow down significantly, and increase contact time, upon encountering a TCR signal (TCR stop signal) (160, 163). Importantly, the stop signal required for the slowing down of Tregs is CTLA-4-independent, as CTLA-4-deficient TCR-transgenic Tregs slowed down as efficiently as CTLA-4-sufficient cells in mixed culture with antigen-pulsed DC (160). Similarly, addition of blocking CTLA-4 antibody to Tregs on lipid bilayers containing CD80, pMHC, and ICAM-1 did not affect their motility (163). Although active displacement of CD28 from the IS by CTLA-4 precludes an active role for the former in the establishment and stabilization of Treg–APC synapses, a growing body of data indicates that CD28 may be important for the orchestration of Treg motility and contact half-life with APC prior to mature synapse formation. However, data on involvement of CD28 in stop signaling remain contradictory. While CD28-deficient Tregs stop normally in mixed culture (160), CD28-blocking antibody interfered with the stop signal (163). Specific loss of CD28 in Tregs reduces surface expression of CTLA-4 on these cells and results in reduced suppressive capacity and systemic autoimmunity (71), indicating that tonic signaling input downstream of transient B7–CD28 interactions may regulate recycling of CTLA-4 protein. CD86 and CD28 input is also important for DC-induced proliferation of Tregs (155).

Unlike Tregs, effector T cells are sensitive to CTLA-4-dependent reversal of the TCR stop signal. In the first report of CTLA-4 dependent reversal of TCR stop signal, Schneider et al. (156) used anti-CTLA-4 stimulation and observed that it enhanced effector T cell motility on LFA-1-coated plates. Moreover, anti-CD3 antibody induced reduction in T cell motility, but a combination of anti-CD3 and anti-CTLA-4 did not elicit this stop signal. In the same study, CTLA-4 expression on effector T cells increased their motility and reduced their contact time with APCs in the context of antigen recognition in the lymph node (156). CTLA-4 was also shown to reverse the TCR stop signal in human effector T cell clones *in vitro* (122). Additionally, CTLA-4 antibody treatment enhanced effector T cell motility in the context of an anti-tumor response (158, 159). However, two other 2 photon imaging studies did not report this differential effect of CTLA-4 blockade on regulatory and effector CD4+ T cell populations (157, 164). A study using a mouse model of T cell responses in pancreatic islet grafts reported that CTLA-4 blockade slightly increased motility of both effector and Treg populations, suggesting that CTLA-4 marginally reduces CD4+ T cell motility *in vivo* (157). However, the imaging performed in this study was conducted in an immunoprivileged site (the islet grafts were injected into the anterior chamber of the eye), which could have affected the cellular motility observed. Moreover, the role of TCR signal in the reported effector and Treg motility changes is unknown, as this study observed direct interactions between pancreatic peptide-specific TCR-transgenic CD4 T cells and islet cells, which do not express MHC class II and, thus, cannot present antigenic peptide to T cells. Another study investigating motility of tolerized diabetogenic CD4+ T cells reported no effect of CTLA-4 blockade on T cell motility (164). However, this study did not differentiate between effector and Treg populations, and the effect on CTLA-4 on control, non-tolerized diabetogenic T cells was not reported (164). Overall, the results from these two studies indicate that CTLA-4 has limited effect on motility of self-antigen-specific CD4+ effector T cells, similar to its relatively limited effect on Treg motility. Given that the natural Treg lineage consists of self-reactive T cells (165), this raises an interesting possibility that the role of CTLA-4 in regulation of synapse stability and cellular motility of CD4+ T cells depends on their TCR specificity.

Importantly, the different effects of CTLA-4 blockade on effector T cells and Tregs have also been observed in a recent study using 2 photon microscopy to examine the behavior of the two CD4+ T cell populations in intact lymph nodes (13). CTLA-4 blockade increased Treg motility but decreased effector T cell motility in the presence of antigen, consistent with the proposed role of CTLA-4 in reversal of TCR-induced motility stop in effector, but not regulatory, T cell populations. Anti-CTLA-4 antibody administration increased effector T cell contact time with DCs presenting antigen, but reduced Treg contacts with DCs, strongly suggesting that CTLA-4 has opposing effects on effector and Treg IS stability *in vivo*. However, as CTLA-4 blockade increased the steady-state motility of Tregs, but had no effect on effector T cell motility in the absence of antigenic stimulation, the reduced effector T cell motility and enhanced clustering with DCs after anti-CTLA-4 treatment could be the result of exclusion of Tregs from T cell: DC clusters, rather than a direct effect of CTLA-4 on effector T cells. Interestingly, this study also reported regulatory-effector T cell contacts that were dependent on Treg recognition of B7 expressed on activated T cells (13), suggesting that CTLA-4: B7 interaction plays a role in regulation of T:T cell synapse formation and facilitates Treg-mediated suppression.

The molecular mechanism of CTLA-4-dependent regulation of effector T cell synapse stability and cellular motility is unknown. The initial observation that anti-CTLA-4 treatment enhances effector T cell motility on LFA-1 coated slides and in response to TCR signal was originally interpreted as evidence for an as yet unidentified CTLA-4-induced signal overriding the TCR stop signal (156). CTLA-4 ligation was shown to reduce IS stability (122, 166) and decrease cytoskeletal rearrangements at the synapse (166) through an unknown molecular mechanism. CTLA-4 ligation was also shown to activate the small G protein Rap1 (167, 168), and CTLA-4-induced Rap1 activity was linked to destabilization of the IS (53). However, CTLA-4 mediated increase in Rap1 activity has also been linked to enhanced LFA-1 mediated adhesion (167–169), which is difficult to reconcile with the reduced synapse stability. Moreover, an *in vivo* study reported that intact anti-CTLA-4 antibody and its Fab fragments enhanced effector T cell motility equally well, suggesting that CTLA-4-dependent signaling did not play a role in the motility enhancement (158). Given the role of CD28 in regulation of cytoskeletal dynamics, it is plausible that CTLA-4 may reduce synapse stability and enhance T cell motility through counteracting CD28-mediated cytoskeletal rearrangement through competition for B7 ligand binding. However, there is conflicting evidence to support this hypothesis. It has been reported that the CTLA-4-dependent increase in motility does not require CD28 expression (160), and that the cytoplasmic region of CTLA-4 is required for regulation of T cell motility (166), suggesting a role for as yet unidentified CTLA-4-dependent signaling.

## Conclusion

B7 ligand recognition plays an important role in orchestrating the IS architecture in both effector T cells and Tregs. During recognition of antigen, B7 ligand binding induces CD28 localization to distinct TCR^low^ clusters within the central region of the effector T cell IS. This CD28 recruitment can be counteracted by CTLA-4 through competition for ligand binding and/or by removal of co-stimulatory ligands through trans-endocytosis. CD28 recruitment to the IS induces PKC-θ localization to the center of the IS, through interactions between PKC-θ V3 hinge region and the proline-rich motif on the cytoplasmic tail of CD28. CD28 ligation leads to cytoskeletal rearrangements at the IS through CD28-dependent control of multiple pathways regulating cytoskeletal dynamics: Vav1 and cofilin activation, filamin A binding, and regulation of actin-uncapping proteins. CD28-dependent PKC-θ recruitment and modulation of cytoskeleton plays a critical role in regulation of effector T cell functions. CTLA-4 is a negative regulator of effector T cell functions, and there is evidence that CTLA-4 can reduce effector T cell IS stability through reversal of the TCR-induced stop signal.

While signaling through CD28 is important for steady-state homeostasis, motility, target recognition, and division of Tregs, their activation results in active exclusion of CD28 and PKC-θ and recruitment of CTLA-4 and PKC-η to the synapse. Both phenomena are required for the suppressive function. In contrast to conventional CD4 T cells, CTLA-4 and PKC-η act as positive regulators of Treg function. Since CTLA-4 binds to B7 proteins with significantly higher affinity than CD28 and exclusively activates PKC-η (152), it is reasonable to conclude that its affinity alone may be sufficient to initiate the exclusion of CD28 from potential B7 binding sites. Preferential activation of PKC-η is then a direct outcome of exclusive CTLA-4 recruitment. Higher affinity of CTLA-4–B7 interactions also explains why Tregs are capable of actively recruiting B7 proteins to the synapse, while effector T cells are not (170). Increased affinity of B7–receptor interaction and recruitment of B7 proteins to the synapse also contribute to more long-lived, stable synapses between Tregs and APCs as compared to conventional T cells.

B7 ligand recognition induces dissimilar immune synapse architecture in mature effector T cells and Tregs, but its role in regulation of the immune synapse dynamics at different stages of T cell development is poorly understood. It remains to be determined if the IS formed by pre-selection thymocytes shows the central localization of CD28 and/or PKC-θ, similar to that observed in effector T cells. Given that immature thymocytes do not show the centralized TCR accumulation at the immune synapse (171, 172), and that PKC-θ is not required for NFκB activation in thymocytes (173), it is likely that CD28/PKC-θ dynamics at the thymocyte IS are different to the dynamics in mature effector T cells. At the other end of the T cell’s lifetime, CD28/PKC-θ immune synapse dynamics in exhausted T cells or T cells from aged individuals are very poorly understood. Anergic murine T cells were shown to display unimpaired PKC-θ recruitment (174), but loss of CD28 from human T cells due to repeated antigen exposure or aging may have implications on PKC-θ synapse localization, resulting in altered kinetics and architecture of the synapse, and changes in downstream signaling. Furthermore, despite the massive amount of data on CTLA-4 biology and the growing importance of the CTLA-4 pathway in immunotherapy, its role in regulation of T cell functions and IS dynamics remains incompletely understood. Since activated effector T cells express CTLA-4 and since surface CTLA-4 is capable of B7 extraction from target membranes regardless of cell type (146), it will be intriguing further to explore the potential role of CTLA-4 in the effector T cell-intrinsic restriction of strength and/or duration of activation, independently of bystander suppression by Treg-expressed CTLA-4. At the signaling level, given the role of CD28 signaling in regulating cytoskeleton dynamics at the IS, CTLA-4 can likely counteract these CD28-mediated pathways, either indirectly through reducing levels of B7 proteins on APCs, or directly through interactions yet unidentified with other binding partners. PKC-η is a likely candidate, as Tregs lacking PKC-η showed enhanced conjugation with DCs, and the CTLA-4–PKC-η complex has been shown to interact with the focal adhesion complex components PAK2 and GIT2, as well as with the guanine nucleotide exchange factor αPIX (152), with a known role in regulating cytoskeletal dynamics (175). Better understanding of the effect of B7 ligand recognition on the IS dynamics at different stages of T cell development and in different T cell subsets is likely to have significant implications for the development of novel immunotherapy strategies.

## Author Contributions

VR and JB wrote the initial draft of this review. VR, NG, and JB rewrote and edited the article.

## Conflict of Interest Statement

The authors declare that the research was conducted in the absence of any commercial or financial relationships that could be construed as a potential conflict of interest.
